# Individualization of Radionuclide Therapies: Challenges and Prospects

**DOI:** 10.3390/cancers14143418

**Published:** 2022-07-14

**Authors:** Hanna Piwowarska-Bilska, Sara Kurkowska, Bozena Birkenfeld

**Affiliations:** Department of Nuclear Medicine, Pomeranian Medical University in Szczecin, 70-204 Szczecin, Poland; sara.kurkowska@pum.edu.pl (S.K.); birka@pum.edu.pl (B.B.)

**Keywords:** radioligand therapy (RLT), theranostics, internal dosimetry, personalized radioisotope therapy, molecular radiotherapy, radiopharmaceuticals

## Abstract

**Simple Summary:**

Currently, patient-specific treatment plans and dosimetry calculations are not routinely performed for radionuclide therapies. In external beam radiotherapy, it is quite the opposite. As a result, a small fraction of patients receives optimal radioactivity. This conservative approach provides “radiation safety” to healthy tissues but delivers a lower than indicated absorbed dose to the tumors, resulting in a lower response rate and a higher disease relapse rate. Evidence shows that better and more predictable outcomes can be achieved with patient-individualized dose assessment. Therefore, the incorporation of individual planning into radionuclide therapies is a high priority for nuclear medicine physicians and medical physicists alike. Internal dosimetry is used in tumor therapy to optimize the absorbed dose to the target tissue. The main reasons for the difficulties in incorporating patients’ internal dosimetry into routine clinical practice are discussed. The article presents the prospects for the routine implementation of personalized radionuclide therapies.

**Abstract:**

The article presents the problems of clinical implementation of personalized radioisotope therapy. The use of radioactive drugs in the treatment of malignant and benign diseases is rapidly expanding. Currently, in the majority of nuclear medicine departments worldwide, patients receive standard activities of therapeutic radiopharmaceuticals. Intensively conducted clinical trials constantly provide more evidence of a close relationship between the dose of radiopharmaceutical absorbed in pathological tissues and the therapeutic effect of radioisotope therapy. Due to the lack of individual internal dosimetry (based on the quantitative analysis of a series of diagnostic images) before or during the treatment, only a small fraction of patients receives optimal radioactivity. The vast majority of patients receive too-low doses of ionizing radiation to the target tissues. This conservative approach provides “radiation safety” to healthy tissues, but also delivers lower radiopharmaceutical activity to the neoplastic tissue, resulting in a low level of response and a higher relapse rate. The article presents information on the currently used radionuclides in individual radioisotope therapies and on radionuclides newly introduced to the therapeutic market. It discusses the causes of difficulties with the implementation of individualized radioisotope therapies as well as possible changes in the current clinical situation.

## 1. Introduction

Nuclear medicine uses radiopharmaceuticals, which are various molecules labeled with radioactive isotopes, for diagnosis and therapy. Radiopharmaceuticals are sources of radiation, and when introduced into the patient’s body (by injection, oral administration, or inhalation), they target specific organs, tissues, or cells. Subsequently, the activity of radiopharmaceuticals in tissues decreases due to their elimination from the body and radioactive decay. Administration of the same activity of a given radiopharmaceutical to different patients can distribute in their bodies differently, and therefore it is important to consider each patient individually. The determination of the total number of nuclear disintegrations that occur in a particular organ allows calculating the mean energy absorbed per kilogram of tissue. This parameter is known as the mean absorbed dose.

The knowledge of the absorbed ionizing radiation dose after administration of a radioactive preparation is of great importance both for the patient’s safety and for the proper course of diagnostics or radioisotope therapy. The activity of radioisotopes, administered to patients for diagnostic imaging studies, must ensure the correct image quality while minimizing the dose that will be absorbed. Due to the increase in sensitivity of modern gamma cameras, the reported diagnostic activities are low. However, in the case of radioisotope therapy, the activity of the therapeutic radioisotope should be as high as possible to effectively destroy tumor cells, and at the same time, low enough not to damage critical organs. Therapy using radioactive isotopes is an extremely important and rapidly developing part of nuclear medicine. Modern radioisotope treatments are based on the idea of theranostics [1,2], according to which a diagnostic examination should be performed with the use of a radiopharmaceutical with the same distribution as the therapeutic radiopharmaceutical. Only when the result of the primary examination shows a sufficiently high accumulation of diagnostic radiopharmaceutical is the patient eligible for the treatment procedure.

In every clinical situation that requires the administration of a radioactive substance to the patient, it is important to know the absorbed dose. Moreover, it is of special importance when the activity is very high, as is the case with radioisotope therapies. Individualized therapy plans, created based on images of a given patient, allow for the optimization of therapy and the minimization of toxic effects [3,4,5,6,7].

Both nuclear medicine and external beam radiotherapy (EBRT) use ionizing radiation to treat malignant tissue. EBRT requires advanced equipment that shapes the external beam to conform to the tumor, and nuclear medicine uses radiopharmaceuticals that are introduced directly into the body. Both treatment techniques should follow the guidelines contained in COUNCIL DIRECTIVE 2013/5/EURATOM from 5 December 2013, concerning the safety of patients diagnosed and treated with ionizing radiation [8]. In Article 56 of the Directive, the following is written: “For all medical exposure of patients for radiotherapeutic purposes, exposures of target volumes shall be individually planned, and their delivery appropriately verified taking into account that doses to non-target volumes and tissues shall be as low as reasonably achievable and consistent with the intended radiotherapeutic purpose of the exposure.” This implies the necessity to personalize the treatment, i.e., the selection of the suitable pharmaceutical, in the right dosage and time. Individual EBRT planning is a common practice that has been developed and used for many years. Teams of physicists involved in treatment planning and clinical dosimetry for each and every patient are the standard in radiotherapy centers. Radiation treatment planning is performed with the use of advanced computer programs using computed tomography (CT), magnetic resonance (MR), or positron emission tomography (PET) images. Modern planning methods include the three-dimensional (3D) technique, which allows for the spatial shaping of radiation beams and the protection of critical organs [9,10].

The situation in nuclear medicine is completely different. Few physicists work in nuclear medicine departments, and radioisotope therapies are usually performed according to standard clinical procedures. Individual calculations of radiopharmaceutical doses for patients are not routinely performed in most nuclear medicine facilities across the world. Nuclear medicine specialists most often use standard activities of radiopharmaceuticals during therapy, considering the patient’s weight or body surface area.

In some cases, administration of standard radiopharmaceutical activities does not provide a sufficiently high dose per tumor to destroy it. On the other hand, giving too much activity could have harmful effects on critical organs. A small fraction of patients receives optimal activity, while the vast majority receive lower doses. This conservative approach provides “radiation safety” to healthy tissues, but also delivers a lower dose than indicated to the neoplastic tissue, resulting in a low response rate and a higher rate of disease relapse. Individualized treatment planning would provide higher absorbed doses to most patients without risking toxicity. “Personalized dosimetry is a must for appropriate molecular radiotherapy”—this is the title of the article by Stabin (one of the pioneers of internal dosimetry) et al. published in 2019 in the *Medical Physics* journal [11]. In this article, the authors present the roots of the difficulties in introducing radionuclide therapy dosimetry (radioisotope treatment planning dosimetry) into routine clinical practices.

## 2. Radionuclides for Therapies

Due to the intensive development of pharmacology, the number of new radiopharmaceuticals that can be used in therapy is increasing every year. A particular advantage of radioisotope therapies is that they can be used in situations where all other forms of treatment have failed. For example, radiopharmaceuticals are administered locally to destroy brain tumors that cannot be surgically removed, and they can be used to treat neuroendocrine tumors spread throughout the body that are refractory to standard chemotherapeutic treatments. Most radionuclides used in therapy emit β− particles, and rarely α particles, which are highly potent. Table 1 contains information on the radionuclides used in radioisotope therapies. Table 2, on the contrary, presents the radionuclides currently being tested, which provides the evidence of the intensive development of this method of treatment.

## 3. Therapeutic Effects of Implemented Internal Dosimetry

In many published scientific papers, the authors have shown a close relationship between the absorbed dose of ionizing radiation and the therapeutic effect in various types of cancer. Strigari et al. [3] evaluated 79 papers that contained dosimetric calculations and the relation of the absorbed dose and the therapeutic effect was found in 48 of them. Even if a significant correlation was found in many articles regarding different radioisotope treatments, there is still a need for prospective clinical trials with many participants. Below, we describe some currently well-established clinical benefits from applied dosimetric calculations.

### 3.1. Treatment of Liver Malignancies with Microspheres

One of the most prominent examples is the use of dosimetry in the treatment of liver malignancies with microspheres (^90^Y glass or resin microspheres). When the activity to be injected is based on dosimetric evaluations, there are high clinical success rates for safe and effective therapy [52,53,54,55,56]. Microspheres are permanently implanted in the tumor via the hepatic artery, which means that there is no biological clearance and, therefore, the calculation can be obtained from a single tomographic scan. Prior to the treatment, simulation with ^99m^Tc-macroaggregates of albumin enables to optimize therapeutic effects by predicting the absorbed dose to lesions and non-tumoral liver. It allows to choose the activity of a single treatment that is effective and safe, and, usually, multiple administrations are not performed. The multivariate analysis of radioembolization in hepatocellular carcinoma presented by Garin et al. in 2017 [57] showed that the dose absorbed by the lesion (larger than 205 Gy) was the only factor associated with the increase of overall survival. It is still discussed whether patient dosimetry should be based on efficacy thresholds or on toxicity thresholds. Chiesa et al. stated that dosimetric prediction for non-tumoral liver is more accurate than lesions [58]. They determined a safety cut-off for non-tumoral liver at 50 or 90 Gy—for bilirubin more or less than 1.1 mg/dL.

### 3.2. Radioiodine Therapy of Thyroid Cancer

Differentiated thyroid cancer is carcinoma deriving from the follicular epithelium and it consists of the majority of thyroid carcinomas. Radioactive iodine therapy can be used after thyroidectomy as an adjuvant therapy for eradication of thyroid remnants or, in patients with advanced metastatic disease, as a curative or palliative treatment. In terms of radioiodine therapy of thyroid cancer, there are two dosimetric approaches: pre-therapeutic and peri-therapy dosimetry [59]. The former aims to assess the activity prior to the treatment, which allows ablation of the remaining malignant tissue and avoids reaching a predetermined threshold for absorbed doses in organs at risk, and the latter assess doses after the treatment, which can help to navigate the number of administrations. Since the treatment of thyroid cancer has been used for decades, attempts have been made to improve its efficacy. The optimal radioactivity of iodine ^131^I administered in the treatment of differentiated thyroid cancer has been controversial since its first use in the 1950s. It has been shown that there are clinical benefits of applied dosimetry in [60,61,62]. Dosimetry-guided radioactive iodine ^131^I treatment allows administration of the maximum possible absorption dose to achieve the maximum therapeutic benefit. The maximal safe dose calculated based on bone marrow irradiation provides an effective means of treatment in patients who failed to adequately respond to conventional fixed-dose therapy. Dosimetric methods of determining ^131^I activities for the treatment of recurrence or metastasis in differentiated thyroid carcinoma are based on the estimation of the ^131^I uptake in tumor or blood. Jin Lee et al. reported that 40–50 Gy delivered to a metastatic lesion is likely to be effective [61]. In the case of the safety approach, which aims to decrease the likelihood of an adverse bone marrow effect, the absorbed dose to blood has been reported to be 2 Gy [60].

There is also a growing number of studies concerning the use of ^124^I PET/CT, which is more sensitive in detecting metastatic disease than ^131^I SPECT/CT, for lesion dosimetry [63,64].

### 3.3. Peptide Receptor Radionuclide Therapy of Neuroendocrine Tumors

Peptide receptor radionuclide therapy (PRRT) is a method of treatment of metastasized or nonresectable neuroendocrine tumors (NETs) that uses labeled somatostatin analogues ([^90^Y]Y/[^177^Lu]Lu-DOTATOC or [^177^Lu]Lu-DOTATATE). The results of the randomized phase III NETTER-1 trial, published in 2017, demonstrated the efficacy of PRRT in patients with NETs [28]. Some papers present the significant correlation between absorbed dose and tumor reduction [65,66]. Based on the literature analysis performed by Cremonesi et al. [67], dosimetry results in PRRT reveal large interpatient variability in absorbed doses to normal organs and tumors, emphasizing the need for personalized dosimetry. The toxicity of the standard four-cycle therapy is not high [68], and therefore there is the potential to increase the number of cycles and, by doing so, increase the clinical effect of the therapy. Sandstrom et al. found that 2–10 cycles of [^177^Lu]Lu-octreotate could be administered before the upper dose limit for the individual patient is reached [69]. During PRRT, the main organs at risk are the kidneys and the bone marrow [70,71,72]. The related toxicity is the major factor limiting the number of treatment cycles administered. It was already found in 2005 by Barone et al. [7] that radiation nephrotoxicity after [^90^Y]Y-DOTATOC therapy is dose-dependent. Garske-Román et al., in the prospective observational study of 200 patients treated with [^177^Lu]Lu-DOTA-octreotate, investigated the impact of a dosimetry-guided study protocol on the outcome and toxicity [73]. Each cycle had 7.4 GBq and they were repeated until the absorbed dose reached 23 Gy to the kidneys, 2 Gy to the bone marrow, or there were other reasons for stopping the therapy. They found that kidney dosimetry predicts patient outcome—patients in whom the absorbed dose to the kidneys reached 23 Gy had longer overall survival than those in whom it did not.

### 3.4. PSMA-Based Therapy of Metastasized Castrate-Resistant Prostate Cancer 

[^177^Lu]Lu-labeled PSMA ligands are a novel therapy for progressive metastasized castrate-resistant prostate cancer (mCRPC) [74]. Prior to the treatment, imaging with [^68^Ga]Ga-PSMA PET/CT is necessary, which enables accurate detection of lesions. There are many published studies that estimate the absorbed dose for tumor and organs at risk (kidneys, salivary glands, lacrimal glands, liver, bone marrow) [75,76,77,78,79,80,81,82,83]. Most of the data show high variability and the need for dosimetric calculations. Calculated radiation-absorbed doses per GBq in case of organs at risk were reported as 0.72–0.88 Gy/GBq for kidneys, 0.21–1.17 Gy/GBq for parotid glands, and 0.02–0.03 Gy/GBq for bone marrow [80,82,83]. Therefore, the critical absorbed dose reported for the kidneys (23 Gy) was not reached in any of these studies. Moreover, in most cases, the organ-absorbed doses did not differ significantly between cycles. Okamoto reported that tumor lesions received a mean absorbed dose per cycle of 3.2 ± 2.6 Gy/GBq (range, 0.22–12 Gy/GBq) [80]. However, doses absorbed by tumor lesions gradually decreased in each cycle [80]. Accumulated doses for malignant lesions are much higher than those of other organs. In a study performed by Fendler et al. [75], the dose delivered to the tumor was 6–12 times higher than that to critical organs. In a different study, the dose absorbed by parotid glands was higher than that by kidneys [76]. Kabasakal et al. [82] evaluated pre-therapeutic dosimetry and, similar to the others, suggested that dose-limiting organs are the parotid glands rather than kidneys and bone marrow.

### 3.5. Others

Established clinical benefits from applied dosimetric calculations are not limited to the therapies listed in the previous sections. In the case of neuroblastoma treated with [^131^I]I MIBG, it was found that the patients who received a tumor-absorbed dose of ≥70 Gy had a higher partial response rate than those receiving less [84]. For bone pain palliation with [^153^Sm]153Sm-EDTMP, the mean absorbed dose was positively related to tumor volume reduction, and patients who had disease stabilization had received at least a 21 Gy mean absorbed dose in the study of Senthamizhchelvan et al. [85]. When the [^186^Re]Re-HEDP was used, a significant correlation between the whole-body-absorbed dose and hematological toxicity was found in [86]. In patients treated with [^131^I]^131^I-tositumomab for refractory B-cell lymphoma, the equivalent uniform dose correlated with response in the study reported by Dewaraja et al. [87]. They found that at a threshold of 2 Gy, both mean absorbed dose and equivalent uniform dose had a positive predictive value for a partial and complete response.

## 4. Why Are the Vast Majority of Nuclear Medicine Therapy Prescriptions Still Not Patient-Specific Dosimetry-Based?

For a nuclear medicine therapy to be considered personalized, treatment planning is essential, including the activity chosen individually for a given patient. The first step in individual planning of radioisotope therapy is to perform a series of diagnostic images, which allows visualizing the distribution and measuring how the activity decreased in time in different organs. The next step is to perform dosimetric measurements. It provides information on the degree of uptake of an administered radiopharmaceutical in pathological tissues and critical organs. The obtained dosimetric report is the foundation for planning the maximum activity on tumors, with a safe level of irradiation of critical organs in a given patient. The last step is to obtain a series of images of the patient recorded after the administration of the therapeutic radiopharmaceutical. Post-therapeutic image analysis is used to verify the individual treatment plan and monitor the effects of radioisotope therapy.

Why is internal dosimetry not routinely practiced in most nuclear medicine facilities? Dosimetric imaging and analysis are technically challenging. Some radionuclides used in therapy are difficult to image with a gamma camera. In such a case, to take a series of diagnostic and dosimetric images, the patient should be administered an appropriate radiopharmaceutical surrogate, which has the same biodistribution as the therapeutic radiopharmaceutical. There are some well-known theranostic pairs, such as ^123^I and ^131^I or ^68^Ga and ^177^Lu [88], but intensive research is being conducted to find new ones. For example, in 2019, Dos Santos et al. [89] employed ^212^Pb in the PSMA-seeking ligands (CA009 and CA012) and used diagnostic [^203^Pb]Pb-CA012 and its biodistribution for dosimetric calculations, which were then extrapolated to therapeutic [^212^Pb]Pb-CA012.

Another difficulty is given by imaging and performing dosimetric calculations in patients treated with alpha-emitters. Measuring the biodistribution of alpha-emitters in patients is challenging due to the low administered activities and low probability of emission photon energies suitable for imaging, it is not impossible though. For example, Pacilio et al. calculated the absorbed dose to bone metastases after the administration of ^223^Ra with the aid of the [^99m^Tc]Tc-MDP scan, which enabled better lesion delineation [90]. In a different case, when treating mCRPC patients with ^225^Ac, Kratochwil et al. [91] used a series of [^177^Lu]Lu-PSMA scans to generate time–activity curves (TACs), which were then extrapolated to the physical half-life of ^225^Ac.

Taking a series of quantitative diagnostic or post-therapeutic images requires the patient’s availability, additional acquisitions, and therefore, additional work time of the single-photon emission computed tomography/CT (SPECT/CT) camera. The series of recorded scintigraphic images should then be analyzed by trained medical physicists, using dedicated software for internal dosimetry. Commercial dosimetry programs are a significant financial expense for departments and hospitals. The lack of trained medical physicists to perform internal dosimetry is another important problem. In the absence of dosimetry software integrated with the gamma camera, the medical physicist should perform the necessary calibrations for external dosimetric programs. There is still no general recommendation on how to perform quantitative SPECT/CT (QSPECT/CT) calibration and quantification in the best way. A lot of studies showed the need for harmonization of QSPECT/CT scanners across centers [92,93]. Moreover, dosimetric analysis of a series of scintigraphic images requires time-consuming segmentation (contouring) of the patient’s tissues and organs on reconstructed sections through the patient’s body. Measurement uncertainties of the obtained results of absorbed doses are relatively large. It was estimated that with manual segmentation of tissue contours on SPECT images, the measurement uncertainty of the determined amount of the absorbed dose for an object with a volume of 33 mL is as high as 40% and increases exponentially with the decrease in the volume of the measured tissue [94]. 

The weakest links in individual dosimetry are the accuracy of the input data and the suitability of the radiobiological models used. In general, there is a clear distinction between diagnostic and therapeutic dosimetry in a given patient. In some cases, when justified by the physical and biokinetic parameters of radiopharmaceuticals, consideration should be given to including in dosimetric calculations, in addition to therapeutic activity, the activity of the diagnostic radionuclide used for SPECT/CT imaging after or before radioisotope therapy. The new recommendations issued by the International Commission on Radiation Units and Measurements in Report 96 [95] include reporting both therapeutic and diagnostic activities of radiopharmaceuticals administered to a patient.

Table 3 summarizes the most important difficulties in introducing individual planning of radioisotope therapies into routine clinical practice.

Difficulties with the clinical implementation of individual planning of radioisotope therapies were reflected in the publication of the European Association of Nuclear Medicine (EANM) in January this year, entitled “EANM position paper on article 56 of the Council Directive 2013/59/Euratom (basic safety standards) for nuclear medicine therapy” [8]. The EANM strongly encourages fostering research that eventually leads to individual treatment planning for all types of radionuclide therapies. However, if a nuclear medicine facility is unable to routinely perform individual dosimetry prior to treating patients, EANM recommends standard therapeutic activities for most procedures. In the publication from October this year, Flux and Buscombe [96], on behalf of the Officers and Council of the British Nuclear Medicine (BNMS), advocate that radionuclide therapies should have the status held by EBRT, for which radiation dosimetry is an integral aspect of clinical practice. BNMS calls for infrastructural and economic changes in nuclear medicine departments to introduce individual planning of radioisotope therapies.

## 5. Trends in Personalized Internal Dosimetry

Most of the listed dosimetric problems should be solved within the next few years, and intensive work is currently being undertaken on simplifying the internal dosimetry techniques.

The first simplification concerns the number of necessary scans performed on one patient. The question arises if performing multiple patient acquisitions is necessary and how many of them should be carried out for individual dosimetric calculations. Freedman et al. [97] propose to decrease the series of four standard acquisitions to two post-treatment scans for PRRT with [^177^Lu]Lu-DOTATATE. However, the authors emphasize the need for further research, as they are concerned that the methods of estimating absorbed doses based on only two scans would be even more user-dependent and require careful analysis of the volumes of interest (VOIs) in images. In a number of papers, published both many years ago and recently [98,99,100,101,102,103,104,105,106,107,108], the authors propose to limit the number of imaging tests performed for dosimetric purposes to one single SPECT/CT acquisition. It is under debate if such simplified dosimetry, based on one measurement, could work properly at all. From a mathematical point of view, there is a lack of measured data to estimate the dose absorbed in a patient’s tissue. However, missing data can be filled with empiric data on the biokinetics of used radiopharmaceuticals. The single time-point dosimetry method requires knowledge about population averages for tracer kinetic parameters. If the biokinetics of the radiopharmaceutical in the analyzed organ and the shape of the TAC have been previously determined based on studies of a given patient population, only one quantitative measurement of the activity at the time corresponding to approximately 1.5 Teff is sufficient (where Teff is the effective elimination half-life of the radiopharmaceutical from the organ) [101,104]. Accuracy analysis of the absorbed dose estimation showed that, for the vast majority of patients, measurement errors were less than 10% [109]. The solution to underestimated or overestimated measurement results could be the creation of databases on the biokinetics of the radiopharmaceutical used in the population. Therefore, in addition to using results of clinical dosimetry measurements to optimize the treatment of individual patients, the obtained data on the biodistribution of used radiopharmaceuticals should also be used to build pharmacokinetic databases on the biokinetics of various radiopharmaceuticals. This population biodistribution should soon become an integral part of programs for simplified, individual internal dosimetry.

The second simplification aims to overcome the difficulties of accurately segmenting organs in SPECT images. Since they have significantly limited resolution, the VOI containing all the accumulated activity is difficult to outline. Moreover, since it is generally larger than the organ itself, the determination of the VOI based on CT images cannot be used because it would not contain all the activity accumulated in the organ. Based on the assumption that the activity in the critical organ is relatively evenly distributed, it has been proposed to evaluate the cumulative dose in the organ based on a “small” VOI placed inside the organ itself [69,110,111]. Since the positioning of this VOI can be difficult and cause differences in dose assessment, an automatic segmentation method has been proposed that eliminates these difficulties [112].

Computer scientists and physicists are developing more and more universal and accessible software for internal dosimetry [113,114,115,116,117].

Due to the rapid development of radiopharmacy, new radiopharmaceutical surrogates can be expected for therapeutic radiopharmaceuticals, which cannot be imaged with the gamma camera [118,119,120]. Radiation dosimetry assessment is often initiated with measuring biodistribution of new radiopharmaceuticals in small animals. To study the biological distribution of radiopharmaceuticals in the human body, it is necessary to find the biological distribution in the rodent body and then the results can be generalized to humans. Preclinical dosimetry studies using small animals are an indispensable step in the pathway from in vitro experiments to clinical implementation of new radioisotope therapies. A lot of studies have shown the practicality of using animal distribution as a model for estimating the absorbed dose in humans [121,122,123,124,125].

Due to the growing awareness of the importance of dosimetry, the Internal Atomic Energy Agency conducts training in that field for doctors and medical physicists. For several years, The European School of Multimodality Imaging and Therapy (ESMIT) has been operating within EANM, the aim of which is to educate specialists at three educational levels, both online and in-person [126,127]. The Dosimetry Project Group, operating within ESMIT, organizes advanced courses for the practical application of clinical dosimetry in radioisotope therapy. The courses enable direct contact with dosimetry experts, exchange of experiences, and cooperation of clinicians, implementing individual internal dosimetry in nuclear medicine facilities around the world. These activities are also related to the existing need for standardization and harmonization of internal clinical dosimetry tools.

## 6. Summary

Radioisotope therapies not preceded by individual dosimetry constitute treatment of a lower standard than EBRT. Currently, clinical internal dosimetry is carried out mainly in nuclear medicine centers, combining clinical activities with scientific research. Therefore, the implementation of the standard procedure for routine, individual dosimetry in nuclear medicine is a priority in that field.

What could chiefs of departments do to implement internal dosimetry for individual planning of radioisotope treatment? Above all, they should employ medical physicists who, due to their education and experience, will make use of the available computational techniques for the preparation of a routine dosimetric calculation tool. Currently, several commercial computer programs for internal dosimetry are already available on the market. Unfortunately, the prices of these programs are quite high. However, due to the very rapid development of information technologies in medicine, there is a high probability that dosimetry programs will soon become an integral part of the standard gamma camera software. 

Rapid progress and development of radioisotope therapies are due to the introduction of new radiopharmaceuticals, which in the future can be used for targeted therapy of other cancers, such as, for example, breast, prostate, and brain tumors. Therefore, new imaging surrogates will also be needed. These surrogates will be used to implement a treatment planning approach to radioisotope therapy with alpha- and beta-emitters, enabling prediction of the absorbed radiation dose and treatment to the maximum tolerated one.

Individual planning of radioisotope therapies has a great chance of becoming a medical standard in the world. To do so, the most crucial aspect is to provide sufficient scientific evidence of the superiority of personalized radioisotope therapy over treatment with standard radionuclide activities.

## Figures and Tables

**Table 1:** Radionuclides used in particular types of radioisotope therapies.

Radionuclide	Basic Radiation Type for Therapy	Chemical and Dosage Form	Indications	Administration Route	References
Iodine ^131^I	β^−^	Sodium iodide	Thyroid carcinoma	Oral	
			Hyperthyroidism		[12,13]
Iodine ^131^I	β^−^		Pheochromocytoma	Intravenous	
		Iobenguane	Paraganglioma		[14,15,16,17]
			Neuroblastoma Carcinoid		
Iodine ^131^I	β^−^	Apamistamab	Leukemia	Intravenous	[18]
Iodine ^131^I	β^−^	Tositumomab	non-Hodgkin’s lymphoma	Intravenous	[19,20]
Iodine ^131^I	β^−^	Lipiodol	HCC, liver metastasis	Intra-arterial infusion	[21,22]
Samarium ^153^Sm	β^−^	Lexidronam	Painful skeletal metastases	Intravenous	[23]
Strontium ^89^Sr	β^−^	Strontium chloride	Painful skeletal metastases	Intravenous	[24]
Yttrium ^90^Y	β^−^	Ibritumomabtiuxetan	non-Hodgkin’s lymphoma	Intravenous	[25]
Yttrium ^90^Y Therasphere	β^−^	^90^Y glass spheres	Unresectable HCC Liver metastasis	Intra-arterial infusion	[26]
Yttrium ^90^Y SIR-Spheres	β^−^	^90^Y resin spheres	Unresectable HCC Liver metastasis	Intra-arterial infusion	[27]
Lutetium ^177^Lu or Yttrium ^90^Y	β^−^	[^177^Lu]Lu-DOTATATE [^90^Y]Y or [^177^Lu]Lu-DOTATOC	Unresectable or metastasized NETs	Intravenous	[28,29]
Lutetium ^177^Lu or Actinium^225^Ac	β^−^ α	[^177^Lu]Lu-PSMA	Prostate cancer	Intravenous	
		[^225^Ac]Ac-PSMA	(mCRPC)		[30,31]
Phosphorus ^32^P Yttrium ^90^Y Rhenium ^186^Re	β^−^	Colloids	Radiosynovectomy	Intra-articular injection	[32]
Radium ^223^Ra	A	Radium dichloride	Painful skeletal metastases	Intravenous	[33,34]

HCC: hepatocellular carcinoma; SSTR2: Somatostatin receptor type 2; PSMA: prostate-specific membrane antigen; NET: neuroendocrine tumor; mCRPC: metastatic castration-resistant prostate cancer.

**Table 2:** Radionuclides currently introduced into radioisotope therapies, at the stage of research.

Radionuclide	Basic Radiation Type for Therapy	Indications	References
Yttrium ^90^Y	β^−^	Breast cancer	[35]
Lutetium ^177^Lu	β^−^	Pancreatic cancer	[36,37]
Iodine ^131^I	β^−^	Neuroblastoma Central Nervous System/Leptomeningeal Metastases	[38]
Phosphorus ^32^P	β^−^	Pancreatic cancer	[39]
Copper ^67^Cu	β^−^	Radioimmunotherapy	[40]
Holmium ^166^Ho	β^−^	HCC, liver metastasis	[29]
Indium ^111^In	Auger e^−^	GEP-NETs, lung and bladder cancer	[41,42,43]
Tin ^117m^Sn	Internal conversion e^−^	Painful skeletal metastases	[44]
Bismuth ^213^Bi	α	Glioblastoma, prostate and bladder cancer	[45,46,47]
Astatine ^211^At	A	Lung cancer, glioblastoma, radioimmunotherapy	[48,49,50,51]

GEP-NET: gastroenteropancreatic neuroendocrine tumor; EC: electron capture.

**Table 3:** Main reasons for the difficulties in clinical implementation of dosimetric imaging and analysis.

**Technical Causes**
Dosimetric imaging and analysis are technically challenging.
Lack of general recommendation on how best to perform QSPECT/CT calibration and quantification.
Lack of integrated, accessible software, which is commercially available (works in progress).
The necessity of multiple patient acquisitions.
Questionable accuracy, uncertainty.
**Other Problems**
Shortage of medical physicists trained and employed to perform internal dosimetry.
Difficulty to image some therapy radionuclides (surrogates needed).
Additional complications with alpha-emitters.
Lack of awareness of healthcare professionals of the increased effectiveness of radioisotope therapies performed using dosimetric calculations.

## Abbreviations

The following abbreviations are used in this manuscript:

SPECT	Single-photon emission computed tomography
QSPECT	Quantitative single-photon emission computed tomography
PET	Positron emission tomography
CT	Computed tomography
EANM	European Association of Nuclear Medicine
BNMS	The British Nuclear Medicine Society
EBRT	External beam radiotherapy
HCC	Hepatocellular carcinoma
SSTR2	Somatostatin receptor type 2
PSMA	Prostate-specific membrane antigen
NET	Neuroendocrine tumors
GEP-NET	Gastroenteropancreatic neuroendocrine tumors
mCRPC	metastatic castration-resistant prostate cancer
VOI	Volume of interest
TAC	Time–activity curve
PRRT	Peptide receptor radionuclide therapy
Teff	Effective half-life
ESMIT	The European School of Multimodality Imaging and Therapy
